# Advanced practice nurses in primary care in Switzerland: an analysis of interprofessional collaboration

**DOI:** 10.1186/s12912-019-0393-4

**Published:** 2020-01-02

**Authors:** Renata Josi, Monica Bianchi, Sophie Karoline Brandt

**Affiliations:** 1 Department of Business Economics, Health and Social Care, SUPSI University of Applied Sciences and Arts of Southern Switzerland, Manno, Switzerland; 2 Global Health Policy Unit, Social Policy, School of Social & Political Science, University of Edinburgh, Edinburgh, UK

**Keywords:** Advanced practice, Primary care, Ethnography, Multi-professional practice, Nurse roles

## Abstract

**Background:**

The increase in the number of chronically ill patients due to ageing is calling existing models of primary care (PC) into question. New care models have recently been implemented in Swiss PC and involve interprofessional teams. This paper aimed to investigate the practice of interprofessional collaboration between advanced practice nurses, registered nurses, and medical practice assistants within new models of PC in Switzerland using the National Interprofessional Competency Framework.

**Methods:**

An ethnographic design comprising semi-structured interviews and non-participant observations was conducted. Sixteen interviews were conducted with care providers at their PC practice. Interviewees included four advanced practice nurses, two registered nurses, six medical practice assistants, and four general practitioners. Nine other health professionals were subsequently observed in their practice. Interviews and observations were conducted by the first author from February to April 2019.

**Results:**

Our analysis of interview and observational data confirmed that role clarification, team functioning, collaborative leadership, interprofessional conflict resolution, patient-centered care, and interprofessional communication have a significant influence on the interprofessional collaboration among health professionals in Swiss PC. Among these domains, role clarification and team functioning were the most frequently raised issues. Both were found to have the potential to negatively influence and, therefore, hinder efficient interprofessional collaboration within PC.

**Conclusion:**

From the analysis, it emerged that role clarification is crucial for effective interprofessional collaboration within new care delivery models in the Swiss PC context. Our study results may inform international health policymakers and practitioners about six important domains of interprofessional care when implementing new care models. Practical experience with new models of care involving advanced practice nurses and medical practice assistants may also influence the regulation of the scope of practice of these health professionals in Switzerland.

## Introduction

For various reasons, including the increasing number of chronically ill patients and significant advances in medicine in recent times, through which patients are living longer and longer, the demand for primary care (PC) and community care services is on the rise. Meanwhile, patients’ needs are becoming more complex with an increase in multi-morbidity rates. This situation calls into question today’s care delivery models.

In light of the current shortage of general practitioners (GPs), countries such as the Netherlands, Sweden, and the UK have successfully introduced Advanced Practice Nurses (APNs) into interprofessional PC teams to fulfill defined tasks and relieve GPs [1–4].

This study focuses on Switzerland, where APNs’ roles and tasks have been studied in in-patient care settings but, thus far, rarely in the context of PC. Only recently some APNs and medical practice assistants (MPAs) have started to work in PC practices (PCPs) who have implemented new models of care. Those care models involve interprofessional teams in which health professionals assume new roles and tasks. This article aims to investigate the practice of interprofessional collaboration within new models of care involving APNs, registered nurses (RN) without APN education, and MPAs in Swiss PC using an established international framework.

## Background

The Swiss PC sector mainly consists of GPs working in individual private practices. In 2016, around 50% of GPs worked in single practices, while only 10% worked in group practices with other specialists [5]. Such group practices often enable the integration of new professional groups into practices, providing additional care and thus facilitating more integrated and coordinated care. New models of care delivery are needed as, for some Swiss regions, a shortage of GPs is expected [6]. A sufficient recruitment of GPs in the future cannot be guaranteed because too few physicians are trained in Swiss medical schools, and too few young doctors specialise in family medicine [7, 8]. The Swiss Health Observatory has projected that, in order to cope with increasing demand, the GP workforce would have to increase by up to 40% by 2030 compared to 2004 [6]. The shortage of GPs today predominantly affects rural areas in Switzerland, where people already encounter difficulties locating a GP in their area.

Evidence from the US shows that integrating APNs into PC teams can play a central role in alleviating physician workforce pressures and improve the care and management of chronic care needs in the patient community [9–11]. However, the roles and tasks that nurses with advanced roles perform differ widely, depending on the clinical setting and country. In Switzerland today, APNs today work mainly in in-patient care settings, but some APNs have recently started working with advanced roles in PC. The professional roles and competencies of APN are however not yet clarified for the PC setting and, therefore, need to be individually negotiated in every practice. The responsibility for the patient stays with the GP as no clinical protocols for APNs exist yet. Furthermore, APNs are not regulated by law, impeding the further development of the role and the implementation of a clear scope of practice. A further factor that impedes the development of APN roles is remuneration. In other European countries such as the Netherlands or Sweden that have implemented APNs, PC is mainly funded by capitation or a combination of capitation and pay-for-performance measures [12]. By contrast, Swiss PC is almost exclusively funded by a fee-for-service scheme (TARMED). This reimbursement scheme only allows for the reimbursement of services provided by medical doctors which means that PC practices employing APNs need to negotiate with insurance companies on how the services provided by APNs are remunerated.

Unlike APNs, MPAs have since long been an established workforce to perform administrative and coordinative tasks in Swiss GP practices. New care approaches now involve MPAs with advanced competencies in health coaching for chronically ill patients [12–15]. Further education programs for MPAs in chronic care management (CCM) have recently been established. These MPA training courses aim to impart the skills necessary to counsel patients with chronic diseases (e.g., diabetes or coronary heart diseases). However, the educational pathways of APNs and MPAs differ significantly as MPAs are vocationally trained for 3 years, while APNs obtain a master’s degree in nursing sciences at the university level.

**Fig. 1:**
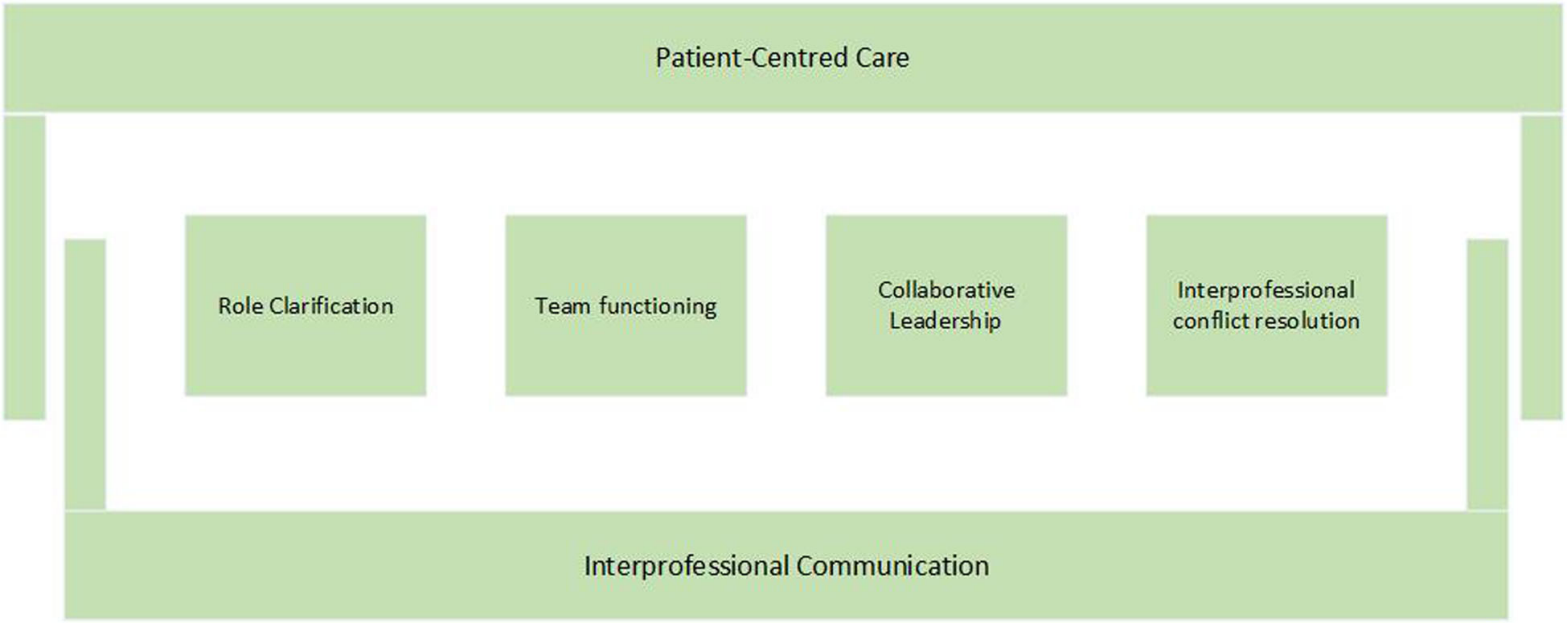
Framework domains of the CIHC National Interprofessional Competency Framework

When newly trained health professionals with newly developed roles are integrated into healthcare teams, interprofessional collaboration (IPC) and collaborative practice are crucial to establishing a well-functioning team that can provide optimal care for patients [16]. International literature found that IPC can improve health outcomes for individuals with chronic diseases, improve patient care and safety, decrease complications, and reduce mortality rates [17]. Furthermore, evidence showed that IPC improves the quality of care [16]. To support the analysis of IPC in diverse contexts, the National Interprofessional Competency Framework (NICF) [18] was created by the Canadian Interprofessional Health Collaborative. The NICF identifies six competency domains for effective interprofessional collaboration (Fig. 1). The four core competencies (role clarification, team functioning, collaborative leadership and interprofessional conflict resolution) are complemented by two domains that support and influence the other domains (patient-centred care and interprofessional communication). The core competencies describe skills, knowledge, attitudes and values needed for effective IPC. Additionally, the way the framework is applied and the way interprofessional collaboration approaches are framed is according to the framework are dependent on three additional considerations: complexity, contextual issues, and quality improvement [18].

As IPC is a major issue which influences how healthcare teams perform within newly composed healthcare settings, and has not yet been studied within Swiss PC, this article presents our research focused on the interprofessional collaboration within new professional roles of APNs, RNs without APN education, and MPAs in Swiss PC using the NICF [18].

## The study

### Design

An ethnographic design [19, 20] involving semi-structured individual interviews and non-participant observations was used for this study. Ethnography focuses on social processes such as behaviours, meanings, and perceptions, as well as the institutional practices that occur within teams, organisations, or communities [21, 22], and was therefore chosen as a method to study the nature and context of new roles of health professionals in PC and to investigate the practice of interprofessional collaboration in this setting.

In the Swiss PC context, in which there are few instances allowing researchers to observe these newly evolving roles and how IPC occurs in practice, ethnography has enabled the detailed investigation of a small number of cases and the interpretation of the meanings and functions of human actions [21]. Semi-structured interviews, in which the sequence of questions is participant-led, have facilitated free-ranging conversations about the research topic [23] and have therefore been considered a reasonable method to capture the views of health professionals on their roles in PC.

Following the semi-structured interviews, non-participant observations were carried out to better understand the health professionals’ working environments and to study how health professionals fulfilled their new roles, how they collaborated within healthcare teams, and how they interacted with patients. For the purpose of this research, non-participant observations in which the subject under research was aware of the researcher’s identity and the research purpose [19] were deemed acceptable to observe health professionals in practice.

### Setting and participants

Contact lists for the recruitment of participants were available through a previous study that the first author had conducted on the skill mix of APNs and MPAs [24]. In this previous study, a convenience sampling strategy was applied to recruit group practices for an online survey in Switzerland. PCPs were included if they operated as group practices with three or more PC doctors. The online survey identified group practices employing APNs, RNs or MPAs. These three groups of health professionals were recruited as potential participants for the present study by the first author by phone or email between December 2018 and January 2019. Potential study participants were informed about the study aim, the design and what their contribution and time effort would be if they participated in the study. As there are very few practices that employ APNs, nurses, or MPAs with specific roles in PC, it was impossible to recruit more participants. All interviews and observations were conducted in the German-speaking part of Switzerland in which the new roles for APNs and MPAs in PC are more established compared to other parts of the country. Interviews and observations were conducted by the first author from February to April 2019. Two APNs, two nurses, and one MPA refused to participate in the study because of time pressures at work or because the institution they worked for did not agree.

### Data collection

#### Semi-structured interviews

In total, 16 interviews with health professionals and GPs were conducted at their respective workplace. Each interview lasted about 35 to 60 min. An interview guide with questions and prompts was created beforehand by the first and second authors. The main study aim guided the development of the interview guide. Authors followed the advice by Braun and Clarke [25] on designing and piloting the interview guide. Interview questions were drafted by the first author and revised by the second author. Through discussion among authors the interview questions were finalised. APNs, nurses, and MPAs were asked about their specific role in the PCP, their clinical tasks, and how their new roles influenced teamwork and the coordination of care. They were also asked about their perceptions of the new roles for health professionals in PC and the possible advantages and disadvantages of the introduction of these new roles. GPs were asked about their perceptions of the new roles of these health professionals in PC and their motivations to employ these health professionals in their practices. Open questions were asked so that participants could freely describe and explain their roles in practice and how they influenced IPC.

With the consent of participants, interviews were audio-recorded and transcribed verbatim. Transcriptions were not returned to participants for checking. To record important thoughts about the interviews to assist the qualitative analysis, the researcher took notes following the interviews.

#### Observations

Non-participant observations, used as an instrument for ethnography [21], were conducted with nine health professionals in order to collect data on the enactment of the roles of APNs, RNs and MPAs in new care models in Swiss PC. Health professionals were observed in their PCP, or outside the practice if they visited patients at home for a short period of 1.5–2.5 h. As the roles of APNs, RNs and MPAs are new to the PC sector, observation data provide important information on how these new roles are enacted in practice shortly after implementation and how IPC functions within these newly composed teams. The observations focused on the tasks fulfilled by the health professionals and whether their statements made during the interviews could be verified in practice. During the observations, notes were taken using an observation scheme and were completed and expanded following the observation. The observation scheme was structured by tasks performed before, during, and after the consultation with the patient. After the completion of the field notes they were critically reflected by the first author. The aim was to assess possible biases in and own feelings towards the observed situation [26, 27].

### Ethical considerations

Prior to starting the study, the Ethics Committee did not require ethical approval, as this study is not covered by the *Swiss Human Research Act* [28]. Nevertheless, written consent was obtained from all participants prior to the interview. All confidential information was protected by removing personal details from the data.

### Data analysis

Transcripts of the interview data, as well as field notes of the observations and observation schemes, were coded by the first and partly by the third author using Atlas.ti 8®. Interview transcripts were coded and then extracted to a processing file to compare and discuss the linked citations of each code or concept among the two researchers. In order to identify themes from the data and to analyse them, authors followed the six-stage framework for thematic analysis proposed by Braun and Clarke [25]. The field notes of the observations were subsequently coded using the concepts and themes identified before. Data from interviews and observations were triangulated and analysed using thematic analysis [29]. Thematic analysis was chosen as data analysis method because of its flexibility in terms of theoretical framework as well as research question [25]. Numerous other studies in the field of nursing have already used thematic analysis for ethnographic data [30–32].

The researchers’ discussion of the concepts and codes derived from the interview and observation data revealed that the concepts found during the analysis should be analysed and compared to the existing literature by using a theoretical framework on IPC. Using an a priori theoretical framework for data analysis allows us to connect research findings to existing literature and to analyse and further develop the research findings [33, 34]. For purpose of analysis we extracted the four competency domains (role clarification, team functioning, collaborative leadership and interprofessional conflict resolution) of the NICF Framework and compared them to our themes and concepts in order to analyse by how far these framework domains apply to the Swiss PC setting and to newly composed healthcare teams in this setting. We have also considered the two supporting domains (patient-centred care and interprofessional communication) in our analysis.

### Coding consistency

The first author coded all data gained through interviews and observations, whereas the third author coded 25% of the total amount of data. This approach serves to establish coding consistency with the first coder [35]. Only minor discrepancies between the two authors’ codes evolved and were resolved by discussion. This approach gave evidence for the consistency of the first author’s codes. For the final analysis, the codes of the first author were used.

## Findings

### Participant and practice characteristics

Interviews were conducted with four APNs, two nurses without an APN education, six MPAs, and four GPs. All participants except for the medical doctors were female. All four APNs, one nurse, and four MPAs agreed to be observed in practice. Participants worked in ten different PCPs. Practice and participant characteristics are summarized in Tables 1 and 2.

**Table 1:** Participant characteristics

	Professional group				Age range	Highest education				Further education			Clinical work experience	Research participation activities	
	APN	Nurse	MPA	GP	Group	Vocational training	Higher vocational training	Master in Nursing Sciences (MScN)	Doctor of Medicine	ANP+ or other DAS	Diabetes and/or nutrition module for MPA	CCM Modules 1&2	yrs	Interview	Observation
GP1				x	4				x				24	x	
GP2				x	4				x				35	x	
GP3				x	5				x				44	x	
GP4				x	2				x				6	x	
APN1	x				2			x		x			18	x	x
APN2	x				4			x		x			32	x	x
APN3	x				4			x		x			33	x	x
APN4	x				4			x					32	x	x
MPA1			x		2	x					x	x	12	x	x
MPA2			x		2	x					x		17	x	
MPA3			x		4	x					x		36	x	x
MPA4			x		1	x					x	x	5	x	
MPA5			x		2	x					x		12	x	x
MPA6			x		2	x						x	14	x	x
N1		x			3		x				x		25	x	
N2		x			4		x						36	x	x

*Notes: Diabetes, nutrition and CCM Modules are offered at different private and public schools for the professional education of MPA in Switzerland*

*Age groups (in yrs): 1: < 30; 2: 31–40; 3: 41–50; 4: 51–60; 5: > 61*

Abbreviations: *APN* Advanced Practice Nurse; *MPA* Medical Practice Assistant; *GP* General Practitioner; *ANP* Advanced Nursing Practice; *DAS* Diploma of advanced studies

**Table 2:** Practice characteristics

Practice #	Practice type		Number of health professionals with specific roles in CCM			Location of practice			Practice type
	Group practice	Solo practice	APN	MPA	Nurse	Canton	Urban	Rural	General practice
1	x		2			ZH	x		x
2	x			1		BE		x	x^a^
3	x		1	1		ZH		x	x
4	x		1	1		SZ		x	x
5	x			1		BE	x		x
6		x			1	BE		x	x
7	x				1	BE		x	x
8	x			1		ZH	x		x
9		x	1			GL		x	x
10	x			1		ZH	x		x

Notes: ^a^general practice with focus on psychosomatic diseases with a high share of chronically ill patients

### Key themes

Concepts and themes relating to IPC were all allocated to the NICF domains and are presented in a synthesized form. Additional file 1: Figure S1 shows the allocation of the themes and concepts to the framework domains. Results regarding the clinical tasks of the different professional groups and organisational issues that arose during the implementation of new roles in PC will be presented in a future article.

#### Role clarification

The three concepts ‘promotion of the acceptance of new roles’, ‘role profile of APN in the GPs view’ and ‘skepticism towards APN in PC’ coming from our data were connected to this competency domain. These concepts included data on how health professionals and GPs see the importance of promoting new roles of health professionals as well as why it is important that roles are clarified within teams and communicated to patients.

All participants stated that establishing their role in the general practice when they started working in the PCP was a difficult process, as these new roles are yet to be fully established in every practice. For health professionals, it is crucial that their new roles are accepted and recognized by patients and by the GPs they work with. How the GPs communicate the new health professional roles to patients is vital to increasing patients’ acceptance of these new roles, as well as to promoting these roles more broadly.

It mainly depends on how I phrase it. (GP2).

It’s most important how the GP communicates with patients, or, umm, only just to say that I exist and that they (patients) will see me; that is, they don’t say “you’re poorly controlled with your blood sugar and you have to see Miss X now” but “you are now allowed to see Miss X because she knows more about it.” (MPA6).

Within health professional practice teams, it is essential that the team members’ roles and competencies are clear to GPs so that they recognize the benefit APNs, nurses, or MPAs can provide to patients and that they refer their patients to them. The competencies and roles of health professionals are clarified through the concepts put into place when the health professionals started working in the practice, as explained by an MPA:

Before I started, we have determined with the GPs which tasks I am allowed to fulfill independently and which not. (MPA1).

Others had to explain their competencies to GPs:

In the beginning, I once presented my work so that they know a bit what the aim of my work is and what are my competencies and what do they (doctors) do, and I have the impression that it helped. (MPA5).

In practices where the roles and competencies of APNs, MPAs, or nurses are less clear to GPs, there is often more skepticism and fewer referrals from GPs to these health professionals.

The role profile of an APN from a GP’s point of view was discussed in two interviews with one GP who works with an APN who described her role as:

At the moment, many things are still discussed with me (by the APN), but I think maybe, most of all, chronically ill patients or follow-up checks with diabetic patients and adjustments can be handled autonomously (by the APN). (GP4).

Another GP, who works with an MPA in his practice, describes his vision of the APN’s role as not focusing on general practice but on community care:

These colleagues that I know who have employed a nurse as APN, I find it the wrong way; for me, an APN should be in the “Spitex” (community care). Nursing belongs to nursing. (GP1).

I would like to rely on a competent “Spitex” where I know they provide the services I would expect from my APN and especially also in further education, coaching, where she should have a certain leading function in Chronic Care so that I can delegate it. (GP1).

These statements show that there are different opinions about which roles health professionals should take on in general practice, and role clarification is crucial for health professionals and GPs to practice effective interprofessional collaboration.

#### Team functioning

This competency domain includes the concepts ‘Motivation of GPs to delegate or not to delegate tasks to the health professionals’, ‘motivation of health professionals to assume new roles’, ‘role of trust in collaborative working’ and ‘support of health professionals among each other”. These concepts contain data on the process of delegation of tasks from the GP to health professionals, the role of trust in this process and how health professionals worked together with each other.

After the inclusion of an APN, a nurse, or an MPA into care teams in the observed PCPs, health professionals and GPs had to adapt to the new composition of the team and to a new distribution of responsibilities. GPs stated that, in the beginning, it was difficult to delegate tasks previously performed by themselves to an MPA or an APN:

The first step was really to let go and to say I’m not the only one who knows everything but that really this is something that could maybe even work better interdisciplinarily. (GP2).

Another GP confirmed:

In the beginning, one controls probably more often but, umm, in the beginning, it was not easy, for sure not. (GP4).

One nurse argued that the reason not to delegate tasks to her could be the doctor’s fear that she would “steal them some work” (N2). Health professionals assumed that it is difficult for doctors to give away full control of their care for the patient:

There are still doctors who want to do everything by themselves and want to bear the full responsibility and know everything about their patient; I do that all by myself. (APN1).

During the observations of APNs and MPAs, only a few occasions of IPC between those two professional groups could be observed. These few occasions illustrate that the collaboration between APNs or MPAs and GPs could be improved by initiating more of these collaboration opportunities. On both occasions, the observations confirmed what the qualitative data analysis had already shown: The clarification of profession-specific roles and team functioning is crucial for effective IPC. But also building trust was crucial for team functioning. For APNs and MPAs, it is crucial that the GPs have trust in their competencies, as described by an APN and an MPA:

They trust that I will refer back to them if I’m not sure and that is, also, there I must ethically take the responsibility for what I’m doing. (APN1).

They give us all a lot of freedom in our practice, and they know very well that when something is not good, that I would give feedback. (MPA1).

On the other side, one GP confirmed:

It is correct that I have to have trust that she is doing it well. (GP2).

After trust was built, many participants gave diverse reasons for GPs to delegate tasks to health professionals. The most important reason was that GPs are relieved from workload pressures through the work of an MPA or an APN. One GP also saw an advantage for the patient:

In my opinion, this is really also a good and useful supplement where I also think that patients are better cared for if one does it like that…I think it can make everything even more interesting if this model develops and we care for patients as a team. (GP2).

For APNs and MPAs, taking on new roles and responsibilities was a challenge but, at the same time, increased their motivation for their work, which, in turn, positively influenced teamwork. Notably, the MPAs regarded their new roles as an opportunity to advance their career:

It is a challenge and an additional motivation to manage something or keep on working (as an MPA) for a longer time. (MPA1).

Some health professionals felt that often tasks that the GPs do not like to perform themselves are delegated. One example is the control of feet in diabetic patients. One nurse described it the following way:

All the tasks that I don’t like, and I don’t have time for, I’m very happy that you are there to do it. And everything else is still under competition. (N2).

In those practices where APNs and MPAs work together, they did not see each other as competitors but supported each other in their work and their new roles.

We never have a discussion on what I should do and what you should do but more ‘could you help me with this?’ (APN1).

In difficult patient situations, the nurse or APN was described as a source of help and advice for the MPA.

In general, teams in the observed practices functioned well because mutual trust was built over time, and the responsibilities of the health professionals were mostly clear to all team members. This enabled task division between GPs and MPAs or APNs who were willing and motivated to take on new tasks and roles. Team functioning was furthermore found to be highly dependent on interprofessional communication, but the observed health professionals were already highly skilled in these areas and therefore functioned well as a team.

#### Collaborative leadership

To this framework domain the concepts ‘dealing with self-responsibility and consultation with the doctor’ and ‘requirements for the implementation of interprofessional teams’ were allocated. They give valuable information on how self-responsibility influenced the work of APN and MPA and what was needed for effective collaboration in practice.

In the observed PCPs, the collaboration between GPs and health professionals was very tight. MPAs and nurses performed most services on the instruction of GPs. Oversight remained with the GP, who defined the specific need of the patient and the goal of the consultation, which was then performed by the MPA or the nurse.

It is clear that I can make a suggestion, or I can say “I would maybe do it like this or that,” but the decision and the prescription how it should happen remains clearly with the doctor. (MPA6).

APNs worked with a high level of responsibility but in close cooperation with GPs. Decisions were often made together, or the APN provided feedback to the GP after consultation with the patient, as described by an APN:

If I can bear the responsibility, I decide by myself but afterward, I inform the responsible doctor. (APN2).

In order to collaborate effectively when decisions were made, for health professionals, it was important that trust was built, competencies were clarified, and GPs could hand over some of the responsibility. One nurse explained it in the following way:

What is important is that everyone knows what he or she is allowed to do, where his or her competencies are and at the end, if this is clarified and it works at the interpersonal level this is all no problem. (N1).

A GP explained further that:

One has to get to know each other well and has to know what she can (the health professional) do on her own responsibility and where am I really needed in addition. (GP4).

#### Interprofessional conflict resolution

This competency domain included the concept named ‘Difficult situations in practice’. Under this concept participants described conflicts that occurred in practice and how they have resolved them.

In the observed PCPs, conflicts were rarely mentioned or observed. The APN stated that tensions in the team sometimes occurred when new GPs or assistants started working in the practice:

If a doctor starts working in our practice, they don’t really know what I can and cannot do and what not and then it happens that they keep controlling me more often. (APN2).

Such conflicts were then resolved through discussion and by showing the doctors or assistants what competencies the APNs have and that they are able to work autonomously.

One nurse said that she initially did not feel welcomed by the team:

…where I recognized that a nurse is not so welcome among the MPA because I can imagine that there are still places (practices) where one clearly still says ‘I do have more competencies than you have’. (N2).

This same nurse resolved the situation by showing the other team members that she wished to collaborate with them and did not want to be seen as someone at a higher hierarchical level.

There appeared to be some potential for conflict when health professionals referred patients to the GP for a further check or examination. In some cases, these patients were not referred back to the health professional to continue consultations after the initial consultation.

And then I realised that I have to see that patients are coming back to me, otherwise then they (doctors) keep them, and I think these are in fact, my patients. (APN2).

#### Interprofessional communication

Interprofessional communication comprised the concepts ‘communication between APN and nurses or MPA’ and ‘communication between GPs and APN, nurses or MPA’. These concepts describe how communication worked in practice and which communication strategies were applied in order to facilitate effective collaboration.

Communication between GPs and health professionals was based on personal dialogue or the electronic health record, which enabled them to indicate if an in-person consultation was not possible during the working day. One MPA said:

Everything we discuss during the consultation the GP is informed about. That means I make good entries in the electronic health record where the GP can trace what we have discussed or what has been done. (MPA2).

If there were urgent questions, it was in most cases possible to immediately ask the GP in the practice, as explained by an APN:

If I have a question, I can go and get the doctor immediately. (APN2).

One GP explained that good interaction between team members was essential:

If we have good contact and we are engaged in conversation and try to find a common language, that is very relieving. (GP1).

In teams where APNs worked together with MPAs, it was important that the APN knew who in the MPA team was in charge of MPAs’ issues in order to communicate and organise task-sharing.

For me, it’s important if I have to know something from an MPA, I know that she (Name) is my contact person because she is the team leader. (APN1).

#### Patient-centered care

To this competency domain the concepts ‘patients’ views on care they receive from health professionals’ and ‘trust from patients towards health professionals’ were allocated. They comprise important information on how patient-centred care is carried out and on how the patients’ view influenced professional practice of health professionals and their collaboration. It furthermore shows, how health professionals think the care provided to patients is seen by patients.

From the participant’s perspective, their care was patient-centered and resulted in positive outcomes for patients. Health professionals believed that their care increased the quality of PC. They also remarked that they felt that patients are not concerned with who is caring for them as long as the quality of care is high, and they feel they are being treated well. An APN said:

I always tell them that I am a nurse and not a doctor and then they always say ‘I don’t care that much’ well, they don’t care that much, they want that someone listens to them and they want to be taken seriously. (APN2).

Another APN added:

I think it has a lot to offer to them, in my opinion, they enjoy optimal care for diabetes and wounds delivered by MPA. (APN1).

Participants also affirmed that they had more time at their disposal for patients, which, in turn, appeared to be much appreciated by patients.

It’s mostly the time I think that one has more time for them, that one listens to them, that they can talk about their issues, I think this is what doctors cannot offer anymore. (MPA4).

Health professionals also observed that their care led to an increase in patients’ self-management.

And if you discuss with patients what they can do themselves, how important it is to take medication if they have to, if you discuss everything in detail, they can do so much, and that serves a lot because they have to come to the practice less often. (MPA4).

MPAs, APNs, and nurses felt that patients trusted them and that the barriers to trust-building were lower for them than for GPs.

One MPA stated:

It’s more that barriers are lower, they can recount more confident things, and they know if I know this, all will be good. (MPA3).

Another MPA added:

Patients dare to ask more, “I’m having this diabetes for ten years but actually, I don’t know what it is or what exactly I have,” or they maybe dare to write an email to me. (MPA6).

## Discussion

We investigated interprofessional collaboration within ten Swiss PCPs, which have implemented new care models involving APNs, nurses, and MPAs in CCM. Our results confirm that the six competency domains defined in the NICF have a significant influence on the practice of interprofessional collaboration. In our sample of newly composed primary healthcare teams, health professionals already had a high level of skill through their education regarding the domains of the framework. We found that role clarification and team functioning were the most frequently raised issues, which in some situations were found to be hindering effective collaborative practice.

The six competency domains of the NICF are influenced by the institutional context and the complexity of care. Unlike other countries such as the Netherlands or Canada, Switzerland does not yet have regulations for the professional practice of APNs or MPAs with new roles in CCM. A lack of regulation negatively influences the role clarification process of health professionals in PC [36]. In the Swiss context, where advanced roles for nurses and new roles in CCM for MPAs are implemented only in a limited number of PCPs in pilot projects, role clarification is a major issue, and it also has a negative impact on team functioning. As only a limited number of practices have so far tried out new models of care, experiences with the implementation of APNs assuming certain tasks of GPs are very limited. When new health professional roles are established, a special effort has to be made by teams and individual professionals to define their roles and to communicate them to others. According to Brault et al. [37], the best performing interprofessional teams are those who have introduced institutional processes to support role clarification. In our sample, those practices who had defined the roles and competencies of the APNs and MPAs before or at the start of the implementation experienced fewer role conflicts after the introduction of the new roles. Furthermore, according to Brault et al. [37], professionals themselves need to make an effort to know their own roles and communicate them to others to support role clarification. Complex care situations, as often the case in chronically ill patients, require a higher number of health professionals working together. This collaboration requires precise role profiles and trust in each other’s competencies. We found that, in complex care situations, tasks were divided according to the competencies of the individual professionals. APNs as well as nurses could support MPAs when challenged by complex care situations. Such a task division according to competencies supports IPC whereby different skills can be combined to optimize patient care.

The analysis of the practice of IPC within a few Swiss PCPs that have implemented new models of care shows that the six competency domains are not easy to attain in practice. When new models of care are implemented in contexts lacking regulations for the professional practice of APNs or MPAs with new roles in CCM, role clarification requires a considerable effort by all team members and takes time. The experience from our sample practices, however, also shows that these interprofessional teams function well and have succeeded in defining the roles of the different professionals so that they can collaborate well and optimize patient care. This could be attributed to the willingness and openness of physicians, as well as other team members, to integrate new professional groups with newly developed roles.

Other European countries such as the Netherlands, Sweden, and the UK have successfully introduced new care models involving nurse practitioners and APN in PC and have documented positive experiences [1–4]. International literature has shown that APN can provide an equivalent or higher level of care compared to physicians in PC [11, 38–40]. Literature also shows positive patient outcomes when MPAs take over the health coaching of chronically ill patients [41, 42] but such new care models involving APN are so far only rarely implemented. In Dutch PCP integrative interdisciplinary care models involving APN and so called ‘Doktersassistenten’ have been widely adopted for CCM [43]. The Dutch ‘Doktersassistenten’ have a similar job profile as MPA with further education in Switzerland [44]. Our findings could also inform these countries on what is needed for PC teams in order to collaborate and work effectively. A new model of care – as implemented in some practices in Switzerland – which combines APNs with new roles for MPAs or nurses in CCM, is unique to the PC sector. This practical experience can help to analyse such new care models and to evaluate the extent to which new care models can be transferred from other countries and adapted to the local health system. Further research should focus on investigating how patients experience these new care models and evaluate how widespread implementation of such models of care can be supported at the health policy level.

### Limitations

There are some limitations to this study. First, we could only recruit a small number of health professionals and GPs. It was impossible to recruit participants outside of the German-speaking part of Switzerland as only recently have some practices implemented new models of care involving these health professionals. Second, we only recruited health professionals who were already employed in PCPs and have taken up advanced or new roles. This selection could influence the results, as practitioners and health professionals who already work in this setting are more likely to be in favor of the implementation of such new care models. Additionally, we collected very few critical statements regarding new models of PC provision.

## Conclusion

This study has deepened the understanding of interprofessional collaboration in the context of newly evolving roles for APNs, nurses, and MPAs in Switzerland. It emerged from our analysis that role clarification is crucial for efficient interprofessional collaboration within new models of care in the Swiss PC context. The experience from our sample practices can inform international health policymakers and practitioners about the importance of the six domains of IPC presented in this article when implementing new models of care. Moreover, the Swiss experience with a care model that combines APN and MPAs or nurses is unique and shows that interprofessional collaboration can be enhanced through such a combination of health professionals. This practical experience with new models of care involving APNs and MPAs may also influence the regulation of the scopes of practice of these health professionals in Switzerland.

## Supplementary information

**Additional file 1: Figure S1.** Allocation of themes and concepts to the NICF framework domains

## Data Availability

The datasets generated and/or analysed during the current study are not publicly available but are available from the corresponding author on reasonable request.

## Abbreviations

APN: Advanced practice nurse
CCM: Chronic Care Management
GP: General practitioner
MPA: Medical practice assistant
MScN: Master in Nursing Sciences
NICF: National Interprofessional Competency Framework
PC: Primary care
PCP: Primary care practice
RN: Registered nurse

## References

[1] Dierick-Van DA. The introduction of the nurse practitioner in general practice. Maastricht: Maastricht University, University Library; 2010.

[2] Drennan VM, Halter M, Brearley S, Carneiro W, Gabe J, Gage H (2014). Investigating the contribution of physician assistants to primary care in England: a mixed-methods study. Health Serv Delivery Res.

[3] van den Driesschen Q, de Roo F (2014). Physician assistants in the Netherlands. J Am Acad Phys Assist.

[4] Lindblad E, Hallman E-B, Gillsjö C, Lindblad U, Fagerström L (2010). Experiences of the new role of advanced practice nurses in Swedish primary health care-a qualitative study. Int J Nurs Pract.

[5] Swiss Health Observatory. Die Hausarztmedizin in der Schweiz – Perspektiven [Internet]. 2016 [cited 2019 Jun 7];Available from: https://www.obsan.admin.ch/sites/default/files/publications/2016/obsan_bulletin_2016-11_d.pdf

[6] Seematter-Bagnoud L, Junod J, Jaccard Ruedin H, Roth M, Foletti C, Santos-Eggimann B. Offre et recours aux soins médicaux ambulatoires en Suisse - projections à l’horizon 2030. Neuchâtel: Observatoire suisse de la santé; 2008.

[7] Hostettler S, Kraft E. FMH Ärztestatistik 2016 [Internet]. 2017 [cited 2019 Jul 15];Available from: https://fmh.ch/files/pdf19/FMH-rztestatistik_2016_korr.pdf

[8] Schweizerische Eidgenossenschaft. Strategie gegen Ärztemangel und zur Förderung der Hausarztmedizin - Bericht des Bundesrates [Internet]. 2011 [cited 2019 Feb 13];Available from: https://www.bag.admin.ch/bag/de/home/berufe-im-gesundheitswesen/medizinalberufe/medizinische-grundversorgung/strategie-gegen-aerztemangel.html

[9] Parker S, Fuller J (2016). Are nurses well placed as care co-ordinators in primary care and what is needed to develop their role: a rapid review?. Health Soc Care Commun.

[10] Sheer B, Wong FKY (2008). The development of advanced nursing practice globally. J Nurs Scholarsh.

[11] Swan M, Ferguson S, Chang A, Larson E, Smaldone A (2015). Quality of primary care by advanced practice nurses: a systematic review. Int J Qual Health Care.

[12] Thom DH, Hessler D, Willard-Grace R, DeVore D, Prado C, Bodenheimer T (2015). Health coaching by medical assistants improves patients’ chronic care experience. Am J Manag Care.

[13] Willard-Grace R, Chen EH, Hessler D, DeVore D, Prado C, Bodenheimer T (2015). Health coaching by medical assistants to improve control of diabetes, hypertension, and hyperlipidemia in low-income patients: a randomized controlled trial. Ann Fam Med.

[14] Sahli R, Jungi M, Christ E, Adrian G. «Chronic Care Management»-Programm in der hausärztlichen Praxis. Swiss Medical Forum – Schweizerisches Medizin-Forum [Internet] 2019 [cited 2019 Mar 15];Available from: https://doi.emh.ch/smf.2019.08055

[15] Sahli R, Jungi M (2017). Chronic Care Management in der Hausarztpraxis.

[16] Martin JS, Ummenhofer W, Manser T, Spirig R (2010). Interprofessional collaboration among nurses and physicians: making a difference in patient outcome. Swiss Med Wkly.

[17] WHO. Framework for Action on Interprofessional Education & Collaborative [Internet]. 2010 [cited 2017 Nov 21];Available from: http://www.who.int/hrh/resources/framework_action/en/

[18] Canadian Interprofessional Health Collaborative (CIHC). A National Interprofessional Competency Framework [Internet]. 2010 [cited 2019 May 21];Available from: https://www.cihc.ca/files/CIHC_IPCompetencies_Feb1210.pdf

[19] Atkinson P, Hammersley M (1994). Ethnography and participant observation. Handbook of qualitative research.

[20] Savage J (2000). Ethnography and health care. BMJ.

[21] Hammersley M, Atkinson P (2007). Ethnography: principles in practice. 3rd ed. London.

[22] Reeves S, Peller J, Goldman J, Kitto S (2013). Ethnography in qualitative educational research: AMEE guide no. 80. Med Teacher.

[23] Kathryn Roulston, Myungweon Choi. Qualitative Interviews [Internet]. In: The SAGE Handbook of Qualitative Data Collection. 1 Oliver’s Yard, 55 City Road London EC1Y 1SP: SAGE Publications Ltd; 2018 [cited 2019 Feb 26]. Available from: http://methods.sagepub.com/book/the-sage-handbook-of-qualitative-data-collection

[24] Josi R, De Pietro C. Skill mix in Swiss primary care group practices - a nationwide online survey. BMC Family Practice [Internet] 2019 [cited 2019 Apr 16];20. Available from: https://bmcfampract.biomedcentral.com/articles/10.1186/s12875-019-0926-7 10.1186/s12875-019-0926-7 PMC6398248 30832589

[25] Braun V, Clarke V (2013). Successful qualitative research: a practical guide for beginners.

[26] Maharaj N (2016). Using field notes to facilitate critical reflection. Reflective Pract.

[27] Phillippi J, Lauderdale J (2018). A guide to field notes for qualitative research: context and conversation. Qual Health Res.

[28] Human Research Act. Human Research Act [Internet]. 2014 [cited 2018 Jun 4];Available from: https://www.admin.ch/opc/en/classified-compilation/20061313/index.html

[29] Braun V, Clarke V (2006). Using thematic analysis in psychology. Qual Res Psychol.

[30] Kennedy HP, Lyndon A (2008). Tensions and teamwork in nursing and midwifery relationships. J Obstet Gynecol Neonatal Nurs.

[31] Zahran Z, Curtis P, Lloyd-Jones M, Blackett T (2012). Jordanian perspectives on advanced nursing practice: an ethnography: advanced nursing practice. Int Nurs Rev.

[32] Gamlen E, Arber A (2013). First assessments by specialist cancer nurses in the community: an ethnography. Eur J Oncol Nurs.

[33] Maxwell J. Designing a Qualitative Study [Internet]. In: The SAGE Handbook of Applied Social Research Methods. 2455 Teller Road, Thousand Oaks California 91320 United States: SAGE Publications, Inc.; 2009 [cited 2019 Sep 16]. page 214–53.Available from: http://methods.sagepub.com/book/the-sage-handbook-of-applied-social-research-methods-2e/n7.xml

[34] Collins CS, Stockton CM (2018). The central role of theory in qualitative research. Int J Qual Methods.

[35] Syed M, Nelson SC (2015). Guidelines for establishing reliability when coding narrative data. Emerging Adulthood.

[36] Heale R, Rieck BC (2015). An international perspective of advanced practice nursing regulation. Int Nurs Rev.

[37] Brault I, Kilpatrick K, D’Amour D, Contandriopoulos D, Chouinard V, Dubois C-A (2014). Role clarification processes for better integration of nurse practitioners into primary healthcare teams: a multiple-case study. Nurs Res Pract.

[38] Dierick-van Daele ATM, Metsemakers JFM, Derckx EWCC, Spreeuwenberg C, Vrijhoef HJM (2009). Nurse practitioners substituting for general practitioners: randomized controlled trial. J Adv Nurs.

[39] Laurant M, Reeves D, Hermens R, Braspenning J, Grol R, Sibbald B. Substitution of doctors by nurses in primary care [Internet]. In: The Cochrane Collaboration, editor. Cochrane Database of Systematic Reviews. Chichester, UK: John Wiley & Sons, Ltd; 2005 [cited 2017 Jul 28]. Available from: http://doi.wiley.com/10.1002/14651858.CD001271.pub2 10.1002/14651858.CD001271.pub2 15846614

[40] Lovink MH, Persoon A, Koopmans RTCM, Van Vught AJAH, Schoonhoven L, Laurant MGH (2017). Effects of substituting nurse practitioners, physician assistants or nurses for physicians concerning healthcare for the ageing population: a systematic literature review. J Adv Nurs.

[41] Bodenheimer T, Willard-Grace R, Ghorob A (2014). Expanding the roles of medical assistants: who does what in primary care?. JAMA Intern Med.

[42] Chapman SA, Blash LK (2017). New roles for medical assistants in innovative primary care practices. Health Serv Res.

[43] Nolte E, Knai C. Assessing Chronic Disease Management in European Health Systemscountry Reports. [Internet]. World Health Organization; 2015 [cited 2019 Aug 1]. Available from: https://www.ncbi.nlm.nih.gov/books/NBK458742/pdf/Bookshelf_NBK458742.pdf

[44] Profil Doktersassistent [Internet]. 2018 [cited 2019 Feb 12];Available from: https://assistentensite.nl/doktersassistent/

