# Supplementary figures and images for: Advanced practice nurses in primary care in Switzerland: an analysis of interprofessional collaboration
Source: BMC Nurs. 2020 Jan 2;19:1. doi: 10.1186/s12912-019-0393-4 (PMC6941298; doi:10.1186/s12912-019-0393-4)

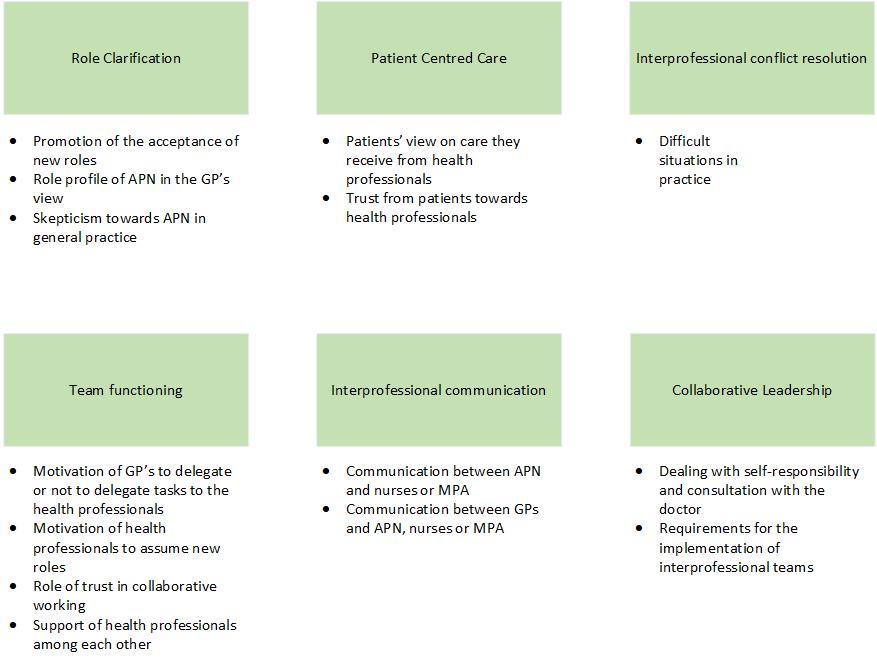

Supplement: Supplementary file 1 — Additional file 1: Figure S1. Allocation of themes and concepts to the NICF framework domains [file 12912_2019_393_MOESM1_ESM.jpg]
